# Vitamin D_3_ Treatment Alters Thyroid Functional Morphology in Orchidectomized Rat Model of Osteoporosis

**DOI:** 10.3390/ijms23020791

**Published:** 2022-01-12

**Authors:** Branka Šošić-Jurjević, Svetlana Trifunović, Jasmina Živanović, Vladimir Ajdžanović, Marko Miler, Nataša Ristić, Branko Filipović

**Affiliations:** Institute for Biological Research “Siniša Stanković”—National Institute of Republic of Serbia, University of Belgrade, Bulevar despota Stefana 142, 11060 Belgrade, Serbia; lanat@ibiss.bg.ac.rs (S.T.); jasminap@ibiss.bg.ac.rs (J.Ž.); avlada@ibiss.bg.ac.rs (V.A.); marko.miler@ibiss.bg.ac.rs (M.M.); negicn@ibiss.bg.ac.rs (N.R.); brankof@ibiss.bg.ac.rs (B.F.)

**Keywords:** vitamin D, thyroid, thyroid-specific proteins, CYP24A1, VDR, rats

## Abstract

Vitamin D plays an essential role in prevention and treatment of osteoporosis. Thyroid hormones, in addition to vitamin D, significantly contribute to regulation of bone remodeling cycle and health. There is currently no data about a possible connection between vitamin D treatment and the thyroid in the context of osteoporosis. Middle-aged Wistar rats were divided into: sham operated (SO), orchidectomized (Orx), and cholecalciferol-treated orchidectomized (Orx + Vit. D_3_; 5 µg/kg b.m./day during three weeks) groups (*n* = 6/group). Concentration of 25(OH)D in serum of the Orx + Vit. D_3_ group increased 4 and 3.2 times (*p* < 0.0001) respectively, compared to Orx and SO group. T_4,_ TSH, and calcitonin in serum remained unaltered. Vit. D_3_ treatment induced changes in thyroid functional morphology that indicate increased utilization of stored colloid and release of thyroid hormones in comparison with hormone synthesis, to maintain hormonal balance. Increased expression of nuclear VDR (*p* < 0.05) points to direct, TSH independent action of Vit. D on thyrocytes. Strong CYP24A1 immunostaining in C cells suggests its prominent expression in response to Vit. D in this cell subpopulation in orchidectomized rat model of osteoporosis. The indirect effect of Vit. D on bone, through fine regulation of thyroid function, is small.

## 1. Introduction

Cholecalciferol (Vit. D_3_) is a secosteroid hormone that is synthesized endogenously in the epidermis from 7-dehydro-cholesterol, induced by ultraviolet radiation from sunlight both in humans and rodent models [1]. It is also found in foods (fatty fish, liver, or egg yolks), and is the most common over-the-counter vitamin D supplement, available in a variety of strengths and forms [2].

The main physiological actions of vitamin D are related to regulation of calcium and phosphorus homeostasis, mainly through direct actions of the hormone in the intestine, kidney, and bone, and through feedback inhibition of PTH production in the parathyroid glands [3,4].

To exert biological activity, Vit. D_3_ needs to be sequentially hydroxylated to calcitriol (1,25(OH)_2_D). The first 25-hydroxylation occurs mainly in the liver, while the second, 1α-hydroxylation occurs mainly in the kidneys [5]. In addition to activating enzymes, renal CYP24A1 seems to be mainly responsible for inactivation and degradation of both 1,25(OH)_2_D and 25(OH)D forms, thus balancing the circulatory concentration of vitamin D [6]. 

In target tissues, the actions of calcitriol are mediated by genomic and non-genomic signaling pathways [5,7,8]. In the genomic signaling pathway, calcitriol binds to nuclear vitamin D receptor (VDR) to regulate transcription of vitamin D target genes. 

1,25(OH)_2_D induce rapid Cyp24a1 gene expression following its binding to VDR receptor [9]. It is expressed not only in the kidney, but also in several other tissues expressing vitamin D receptor, and it plays an important role in local modulation of vitamin D activity [2,9,10]. Higher expression of CYP24A1 in various types of cancer tissues induces local vitamin D insufficiency, thus promoting cancer growth [11].

Vitamin D deficiency, a frequent companion of aging, has been identified as a risk factor for the development of autoimmune and malignant diseases of the thyroid gland [12,13,14,15]. Expression of VDR and vitamin D metabolic enzymes have been confirmed in normal and malignant thyroid tissue [16,17] and in FTRL-5 cells [18]. On the other hand, most immune cells also express VDR and vitamin D metabolic enzymes [19,20]. It is still unclear whether the correlation between vitamin D deficiency and the development of thyroid disease indicates a pathological mechanism in the thyroid gland, a causal relationship, or the correlation is a consequence of a defect or dysfunction of the immune system [20].

Vit. D supplementation is indispensable in the prevention/treatment of osteoporosis and metabolic disorders. Thyroid hormones also play important roles in regulation of bone remodeling cycle and health [21]. There is currently no data in the literature, clinical or experimental, whether and how vitamin D treatment affects thyroid function in the context of osteoporosis. The model of orchidectomized middle-aged rats for male osteoporosis is well-defined by other researchers [22] and in our laboratory [23]. Skeletal effects of orchidectomy were confirmed using different methods—dual energy X-ray absorptiometry (DEXA), X-ray microtomography, and histomorphometry—but histomorphometric examinations confirm the trabecular bone loss more rapidly, 2–3 weeks after orchidectomy [12,22,24,25,26]. Specifically, in our laboratory analyses of trabecular microarchitecture of the proximal tibia metaphysis confirmed marked decrease of cancellous bone area, trabecular thickness, and trabecular number [23]. Serum osteocalcin and PTH levels were elevated, calcitonin was decreased, as well as serum calcium and phosphorus, testosterone was diminished while corticosterone and thyroid hormones remained unaltered in comparison with a sham-operated group [23,27,28,29]. 

The aim of the present study was to investigate the hypothesis that vitamin D treatment affects thyroid morphology and function, as well as immunohistochemical expression of Vit. D catabolic enzyme CYP24A1 and VDR, in our model of middle-aged male osteoporosis.

## 2. Material and Methods

### 2.1. Animals and Treatments 

Male Wistar rats used in the experiment were bred and housed in the Unit for Experimental Animals at the Institute for Biological Research “Siniša Stanković”, National Institute of the Republic of Serbia (IBISS), Belgrade, Serbia. The animals were housed in controlled ambient temperature (21 ± 2 °C) and lighting conditions (12 h light–12 h dark). Food (commercial pellets for rats; Veterinarski Zavod Subotica, Victoria group, Subotica, Serbia) and water were provided ad libitum. All animal procedures were in compliance with the Directive 2010/63/EU on the protection of animals used for experimental and other scientific purposes and were approved by the Ethical Committee for the Use of Laboratory Animals of IBISS, University of Belgrade (no. 01–1321). 

At the age of 15 months, animals were randomly divided into two groups: one was bilaterally orchidectomized (Orx, *n* = 12) via the scrotal route. Animals were intramuscularly injected with ketamine anesthesia (15 mg/kg body mass; Richter Pharma, Austria), 15–20 min prior to orchidectomy. The scrotal area was shaved and cleaned with the antiseptic solution (Octenisept, Schuelke & Mayr GmbH, Norderstedt, Germany). Using a sterile scalpel, scrotum and lamina parietalis were incised in the middle. Since rats have open inguinal canals, testicles were forced into the scrotum from the abdomen as needed. This was performed by exerting gentle pressure towards the scrotum in the caudal abdomen with fingers. Next, the testicular content (both testicles, two epididymides, vasa deferentia, and the testicular blood vessels) was gently exteriorized. Vasa deferentia and blood vessels were ligated with an absorbable surgical suture, and the testicles and epididymides were removed using scissors. The remaining tissue was placed back in the scrotal sac using blunt forceps. The scrotal skin was not sutured. After orchidectomy, the animals were housed individually and kept under close observation for approximately 24 h after the surgery. Considering healing and bleeding, no negative impacts were observed. 

The second group (SO; *n* = 6) was sham-operated, in which testicles were exposed but not removed. Two weeks after the surgery, the treatment begun: one group of animals was subcutaneously treated with 5 µg (200 IU) of cholecalciferol (Orx + Vit. D_3_; Sigma Aldrich, Germany; *n* = 6)/kg b.m. daily, dissolved in sterile olive oil, while two control groups, orchidectomized (Orx; *n* = 6) and SO, received the same amount of vehicle alone for three weeks. 

### 2.2. Sample Collection and Processing 

Animals were decapitated without anesthesia to avoid the possible effects of anesthesia on serum hormone results. 

Blood was collected from the trunk, and the serum stored at −70 °C. After decapitation, the thyroids from each animal were excised and weighed. The relative organ weights were calculated from the ratio of the measured organ weight and body mass for each animal.

For histology, the thyroids were fixed in Bouin’s solution for 48 h and dehydrated in increasing concentrations of ethanol and xylene. After embedding in Histowax (Histolab Product Ab, Sweden), tissue blocks were serially sectioned at 5 µm thickness on a rotary microtome (RM 2125RT Leica Microsystems, Germany). Tissue slices were subjected to hematoxylin and eosin (H&E) staining and immunohistochemistry. 

### 2.3. Transmission Electron Microscopy (TEM)

For transmission electron microscopy (TEM), one thyroid lobe was removed from two randomly chosen animals per group, sliced in 4% glutaraldehyde solution in 100 mM phosphate buffer, pH 7.4, for 24 h at 4 °C, and further processed as previously described [30]. In brief, post fixation was carried out with 1% OsO4 for 1 h at 4 °C, and counterstaining with uranyl acetate. Samples were dehydrated through a graded series of ethanol and embedded in Araldite resin. A Leica EM UC7 ultramicrotome (Leica, Germany) with a Diatome ultra 45° diamond knife (Diatome, Switzerland) was used for cutting ultrathin sections of thyroid tissue at a thickness of 70 nm. Grids with ultrathin sections were stained with uranyl acetate and lead citrate and examined under a Morgagni 268 (FEI Company, The Netherlands) transmission electron microscope.

The number lysosomes in thyrocytes was analyzed on TEM micrographs manually, while their diameter was measured by using Windows based ImageJ (Image J, Version 1.49j). Measurements were conducted on 10 thyrocytes per group.

### 2.4. Immunohistochemistry (IHC) and Immunofluorescence (IFC)

After tissue deparaffinization, endogenous peroxidase activity was blocked by incubation of sections with 0.3% hydrogen peroxide in methanol for 15 min. Then, thyroid sections were exposed to heat-induced antigen retrieval to unmask target antigens. Slides were placed in a container, covered with 100 mM sodium citrate buffer (pH 6.0), and heated in a microwave oven at 750 W for 3 × 7 min. Reduction of nonspecific background staining was achieved by incubation with normal porcine serum (code no. x0901, Dako, Denmark), diluted 1:10 for 45 min. 

Information on antibodies used is summarized in Table 1. For analysis of thyroid-specific proteins, the antiserum directed against human thyroid peroxidase (TPO), thyroglobulin (Tg), and sodium iodide symporter (NIS) were applied overnight at 4 °C (Table 1). For immunodetection of vitamin D-metabolizing enzymes and VDR, antiserum directed against each protein was applied overnight at 4 °C (Table 1). Secondary antibodies, anti-mouse or anti-rabbit HRP-labeled antibodies, were applied for 1 h at room temperature. All washes and dilutions were performed using 0.1 mol/L PBS pH 7.2. 

To confirm that the observed staining is not caused by non-specific interactions of the antibody with the tissue (negative control) in case of VDR and CYP24A1, the primary antibody was substituted with an “irrelevant primary antibody”. Irrelevant primary antibody for this purpose was polyclonal rabbit anti-rat beta-LH (obtained from Dr. A. F. Parlow, National Hormone Peptide Program, Harbor-UCLA Medical Centre, USA). It is not expressed in the thyroid, has the same isotype as the specific primary antibodies (polyclonal rabbit IgG), and was applied at the same concentration. To control the background staining, the primary antibodies were substituted with phosphate-buffered saline (PBS). Parathyroid glands served as the positive control of IHC staining.

Hematoxylin was used as counterstain, and slides were then mounted in DPX medium (Sigma-Aldrich, Barcelona, Spain). Digital images of the thyroid sections were made on a DM RB Photomicroscope with a DFC 320 CCD Camera (Leica, Wetzlar, Germany).

For double-immunohistochemical labeling of calcitonin (CT) and CYP24A1 (Table 1), Tyramide signal amplification kit with HRP–goat anti-rabbit IgG and Alexa Fluor^®^ 568 tyramide (cat. no. T20924; Invitrogen, Waltham, MA, USA) was used according to manufacturer’s instructions. To avoid false colocalization using two rabbit antibodies, we used the microwave treatment described by [31]. In brief, after overnight immunostaining of CT and following incubation with goat anti-rabbit Alexa Flour 488, sections were rinsed extensively in PBS (pH 7.4), blocked in PBS with 1% bovine serum albumin (BSA) for 1 h, and then incubated with thyramide for 10 min. After extensive rinsing in PBS (pH 7.4), the slides were immersed in citrate buffer (pH 6.0) and heated in a microwave oven at 750 W for 7 min. After cooling down, sections were stained for CYP24A1 (Table 1) overnight at 4 °C and visualized using goat anti-rabbit Alexa flour 568. Finally, nuclei were stained with 4′,6-diamidino-2-phenylindole (DAPI; Euromedix, cat. no. 1050-A), by incubating cells with 300 nmol of DAPI dissolved in PBS (1:300) for 5 min. Microscopic slides for immunofluorescence were mounted in Mowiol (Calbiochem, Millipore, Germany) and captured on a Zeiss Axiovert fluorescent microscope (Zeiss, Germany).

### 2.5. Quantification of IHC and Morphometric Analysis

Quantification of IHC signal and morphometric analysis were performed independently by two researchers who were blind to the treatment given to the animals. The stained percentage color area for the DAB immunostaining was evaluated using a Windows based ImageJ (Image J, Version 1.49j) according to previously described procedures [30]. For the analysis of DAB immunopositive follicles, 10 randomly captured images (the Leica light microscopic tool has already been described; 2088 × 1550 pixels, ×40 objective magnification) per thyroid tissue per animal were analyzed.

Morphometric analysis of all abovementioned immunohistochemically stained thyroid sections was carried out as previously described [30]. In brief, for each primary antibody, three sections taken from the central part of the thyroid gland per animal were analyzed (*n* = 6/group). Measurements were carried out using a newCAST stereological software package (VIS–Visiopharm Integrator System, version 3.2.7.0; Visiopharm; Denmark), at an objective magnification of ×40. The counting area was defined using a mask tool; test grid (6 × 6) with uniformly spaced test points and lines was provided by the new-CAST software. Test points hitting the corresponding immunopositive tissue components were determined. The relative volume densities (V_V_) were calculated as the ratio of the number of points hitting the immunopositive tissue component divided by the number of points hitting the reference space, i.e., analyzed thyroid section: V_V_ (%) = Pp/Pt × 100 (Pp, counted points hitting the immunopositive tissue component; Pt, total of points of the test system hitting the reference space, the sum of both immunopositive and immunonegative counts). For Tg-immunostained sections, V_V_ of the immunopositive follicular epithelium and colloid as well as non-reactive interstitium was estimated. 

### 2.6. Hormone Analysis

Serum concentrations of 25-hydroxyvitamin D and total T_4_ were measured using commercially available electrochemiluminescence immunoassay kits (Roche Diagnostics GmbH, Mannheim, Germany) on cobas e 411 and e 601 immunoassay analyzers (Roche Diagnostics), respectively. Concentration of TSH was measured with a commercially available rat TSH ELISA kit (IBL International GmbH, Hamburg, Germany). Serum calcitonin concentration was assayed using commercially available chemiluminescence immunoassay (Nichols, Tioga County, NY, USA) on the MLA-1 chemiluminiscence analyzer (Ciba-Corning, Medfield, MA, USA) All samples were assayed in duplicate together in one run, and results were accepted if the coefficients of variation were <10%.

### 2.7. Statistical Analysis

Statistical analysis of all the obtained results was performed using GraphPad Prism v.8 for Windows (San Diego, CA, USA). After confirmation of normality of distribution (Kolmogorov–Smirnov test) and the homogeneity of variance (Bartlett’s test), the data were analyzed by one-way ANOVA, while the Tukey post hoc test was used to evaluate differences between the groups. A confidence level of *p* < 0.05 was considered statistically significant. The data are summarized as mean ± SD.

## 3. Results 

### 3.1. Thyroid Weight and Body Mass

The results on absolute and relative thyroid weight, as well as body mass are summarized in the Table 2. No difference in absolute thyroid weight or body mass were detected between the experimental groups. However, the observed differences in relative thyroid weight between Orx + Vit. D_3_ and SO group (by 22%, *p* = 0.08), although non-significant, may be worthy of further investigation in subsequent more highly powered studies. 

### 3.2. Serum Hormone Concentrations

**Figure 1:**
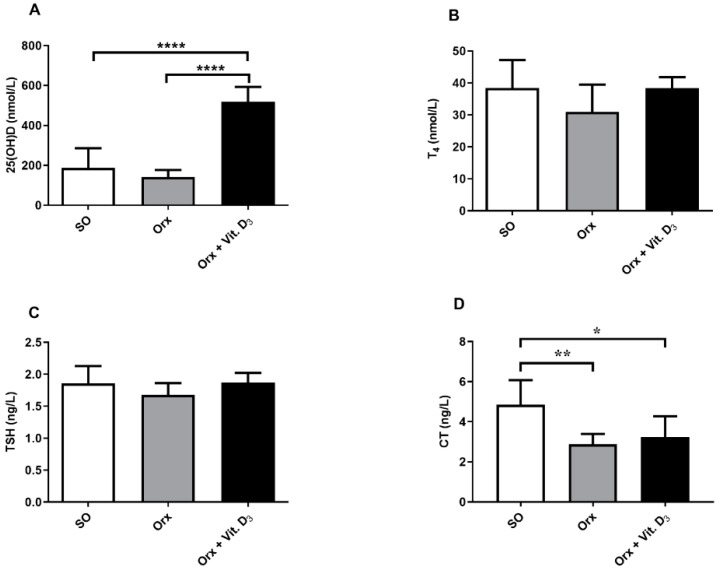
Concentration of total 25-hydroxyvitamin D (25-(OH)D; nmol/L; (**A**), total thyroxine (T_4_; nmol/L; (**B**), thyroid stimulating hormone (TSH; ng/L; (**C**), and calcitonin (CT; ng/L; (**D**) in serum of sham-operated (SO), orchidectomized (Orx), and orchidectomized cholecalciferol- treated (Orx + Vit. D3) rats. The data are mean ± SD, *n* = 6; * *p* < 0.05; ** *p* < 0.01; **** *p* < 0.0001.

The mean serum concentration of 25-(OH)D in serum of SO rats was 159.8 ± 36.8 nmol/L. After treatment of Orx rats with vitamin D_3_, serum 25-(OH)D concentration was 4 times higher (*p* < 0.0001; Figure 1A) in comparison with the concentration obtained for the value obtained for Orx group, and was 3.2 times higher in comparison with the SO group (*p* < 0.0001; Figure 1A). The total T_4_ and TSH levels in serum remained unchanged (Figure 1B,C). Concentration of calcitonin in serum was 40% (*p* < 0.01) and 33% (*p* < 0.05) lower in Orx and Orx + Vit. D_3_ group, respectively, in comparison with the SO group. 

### 3.3. Histological and TEM Analysis of the Thyroid 

**Figure 2:**
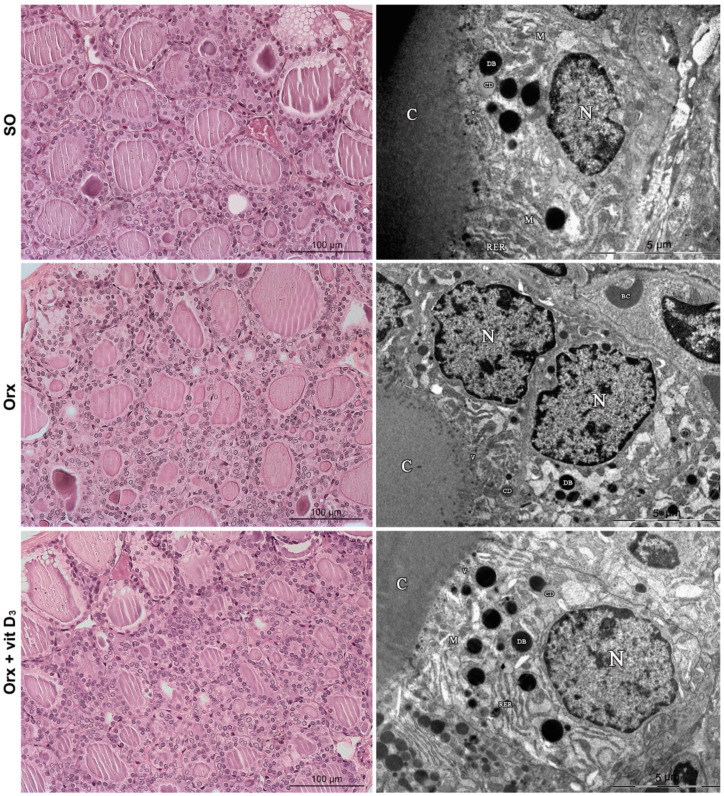
Representative micrographs of hematoxylin and eosin-stained thyroid sections (left column) and transmission electron microscopy of thyrocytes (right column) from sham-operated (SO), orchidectomized (Orx) and orchidectomized cholecalciferol- treated (Orx + Vit. D_3_) rats. N, nucleus; C, colloid; M, mitochondria; RER, rough endoplasmic reticulum; DB, dense bodies, lysosomes; CD, colloid droplets; V, small electron dense vesicles.

The thyroid of all experimental groups is characterized by a regular follicular structure: the follicles in proximity to connective tissue capsule were larger, while more centrally located follicles were of smaller size. However, the thyroid of Orx animals and more notably of Orx + Vit. D_3_ group, was characterized by a microfollicular architecture and follicules containing smaller amounts of luminal colloid in comparison with the SO group (Figure 2, left column).

Electron microscopic examination confirmed that the follicular epithelium was generally cuboidal in all experimental groups, with a large nucleus at the basal pole and more pronounced organelles at the apical pole of the cell (Figure 2, right column). Cisternae of rough endoplasmic reticulum (RER) appeared dilated in all groups. On the other hand, dense bodies–lysosomes were more abundant in Orx + Vit. D_3_ rats (Figure 2, right column), indicating intensified utilization of stored colloid and release of thyroid hormone in the blood stream. The average number of lysosomes per thyrocyte was: 13.64 ± 4.4 for SO, 12.2 ± 3.2 for Orx and 16.6 ± 2.6 for Orx + Vit. D_3_ rats. The average number of lysosomes per thyrocyte significantly increased in Orx + Vit. D_3_ rats compared to Orx rats (by 36%, *p* < 0.01). Lysosomes were heterogeneous in size, their average diameter varied similarly in all experimental groups from 0.2 µm to 1.0 µm. 

### 3.4. IHC Analysis of Thyroid-Specific Proteins

**Figure 3:**
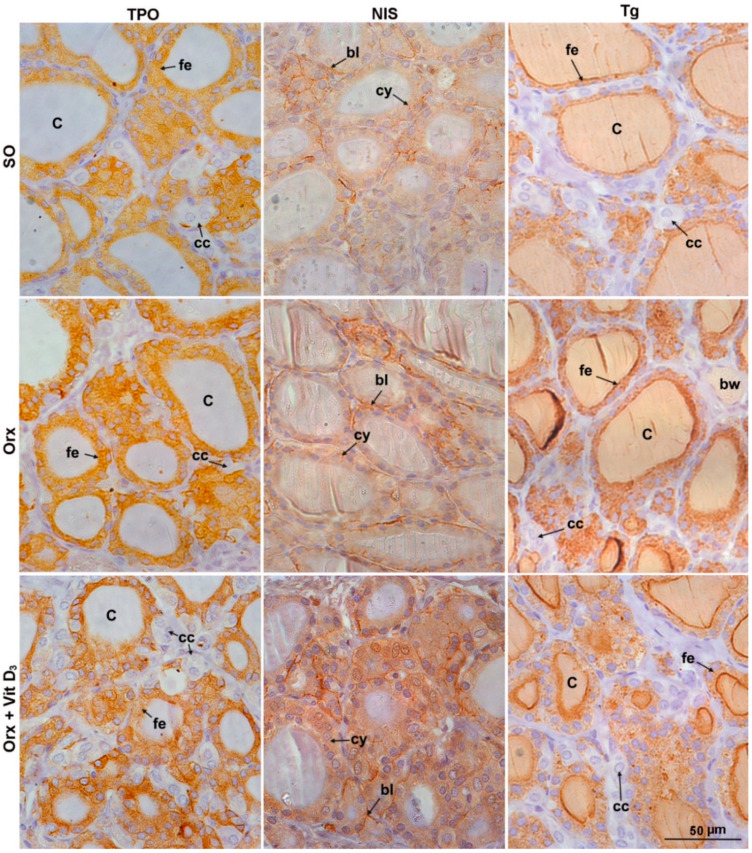
Representative micrographs of immunohistochemical staining showing localization of thyroid peroxidase (TPO), sodium iodide symporter (NIS), and thyroglobulin (Tg) in the thyroid of sham-operated (SO), orchidectomized (Orx) and orchidectomized cholecalciferol-treated (Orx + Vit. D_3_) rats. C, colloid; fe, follicular epithelium; cc, CT-producing cells (C cells); bl, basolateral NIS immunostaining; cy, cytoplasmic NIS immunostaining; bw, blood vessel.

**Figure 4:**
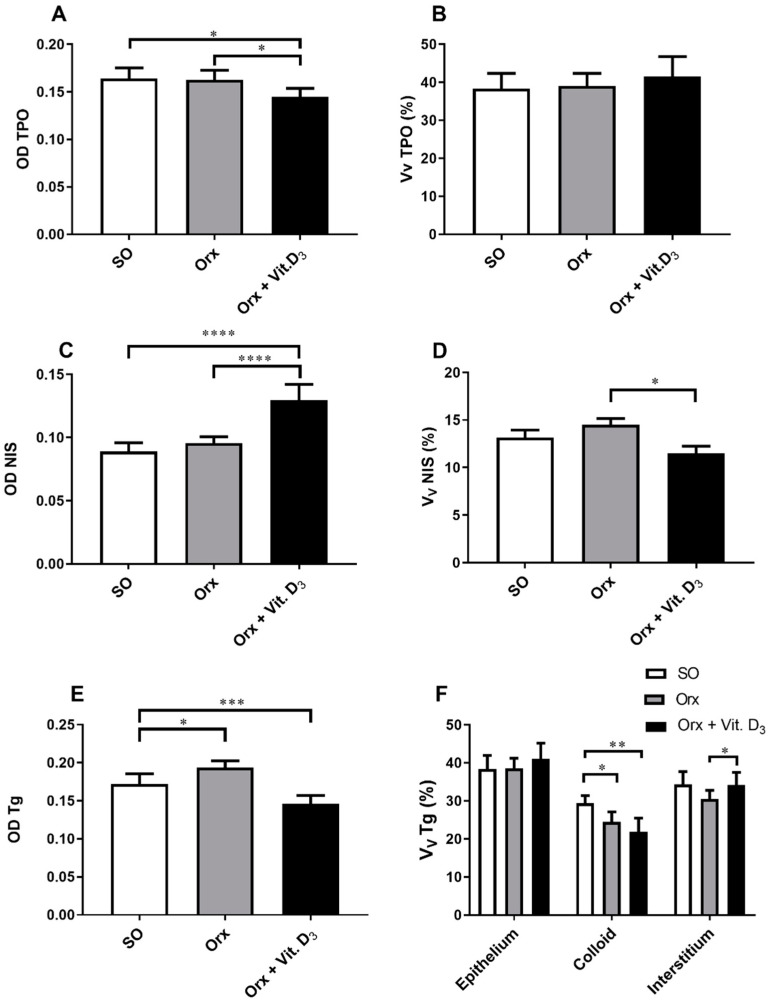
Optical density (OD) of TPO and and the relative volume density of TPO immunopositivity (V_V_ TPO; %) (**A**,**B**), OD of NIS immunopositivity (**C**) and the relative volume density of NIS membrane immunopositivity (V_V_ membrane NIS; %; (**D**), OD of Tg (**E**) and the relative volume density of Tg-immunopositive epithelium and colloid, as well as Tg-negative interstitium (Vv; %; (**F**)) in the thyroid of sham-operated (SO), orchidectomized (Orx), and orchidectomized cholecalciferol-treated (Orx + Vit. D3) rats. All values are means ± SD (*n* = 6); * *p* < 0.05; ** *p* < 0.01; *** *p* < 0.001; **** *p* < 0.0001.

Immunostaining analysis of thyroid-specific proteins showed differences among the experimental groups (Figure 3 and Figure 4). 

The IHC staining pattern of TPO was diffuse and fine granular in the cytoplasm of thyroid epithelium, being more intense at the apical pole of some follicles in all experimental groups (Figure 3). Analysis of OD for TPO revealed that the signal intensity was lower in Orx + Vit. D_3_ and Orx group in comparison with the SO group, by 13% and 12%, *p* < 0.05, respectively (Figure 4A). No significant change in relative volume density of TPO-immunopositive epithelium was detected (Figure 4B). 

NIS, the protein responsible for uptake of iodide, is localized mainly at the basolateral plasma membrane of thyroid epithelium, though the weak cytoplasmic immunostaining was also present in the thyroid of SO rats (Figure 3). The most abundant cytoplasm immunostaining was demonstrated in the Orx + Vit.D_3_ group (Figure 3). The optical density of NIS immunopositivity was 34% (*p* < 0.0001) and 53% (*p* < 0.0001) higher in comparison with OD for Orx and SO groups, respectively (Figure 4C). However, the relative volume density of NIS membrane-immunostained epithelium was 20% (*p* < 0.01) lower in the Orx + Vit. D_3_ group in comparison with the value obtained for Orx rats (Figure 4D). 

Tg is the most abundant thyroid-specific protein synthesized by follicular epithelium, which serves as the substrate for the synthesis of thyroid hormones at the thyrocyte–colloid interface, as well as the storage for inactive thyroid hormones and iodine. Tg immunostaining allows analysis of Tg-immunopositive epithelium (strongest immunodetection) and colloid (weaker immunodetection), as well as immunonegative interstitium (Figure 3). With regard to the colloid, Tg immunostaining displayed follicular heterogeneity—being most prominent in the colloid of small follicles (Figure 3). OD for Tg was lower in Orx + Vit. D_3_ compared to both Orx and SO groups, by 23% (*p* < 0.001) and 15% (*p* < 0.05), respectively (Figure 4E). The relative volume density of Tg-immunopositive epithelium in the thyroid gland was 15% (*p* < 0.05) higher in Orx + Vit. D_3_ group in comparison with the V_V_ epithelium of SO group (Figure 4F). V_V_ of colloid decreased in both Orx + Vit. D_3_ and Orx groups in comparison with SO controls, 26% (*p* < 0.01) and 20% (*p* < 0.05), respectively (Figure 4F). V_V_ of interstitium increased in the Orx + Vit. D_3_ group in comparison with Orx controls by 14% (*p* < 0.01) (Figure 4F). 

### 3.5. Immunohistochemical Expression of CYP24A1 and Vitamin D Receptor (VDR) in the Thyroid Tissue

**Figure 5:**
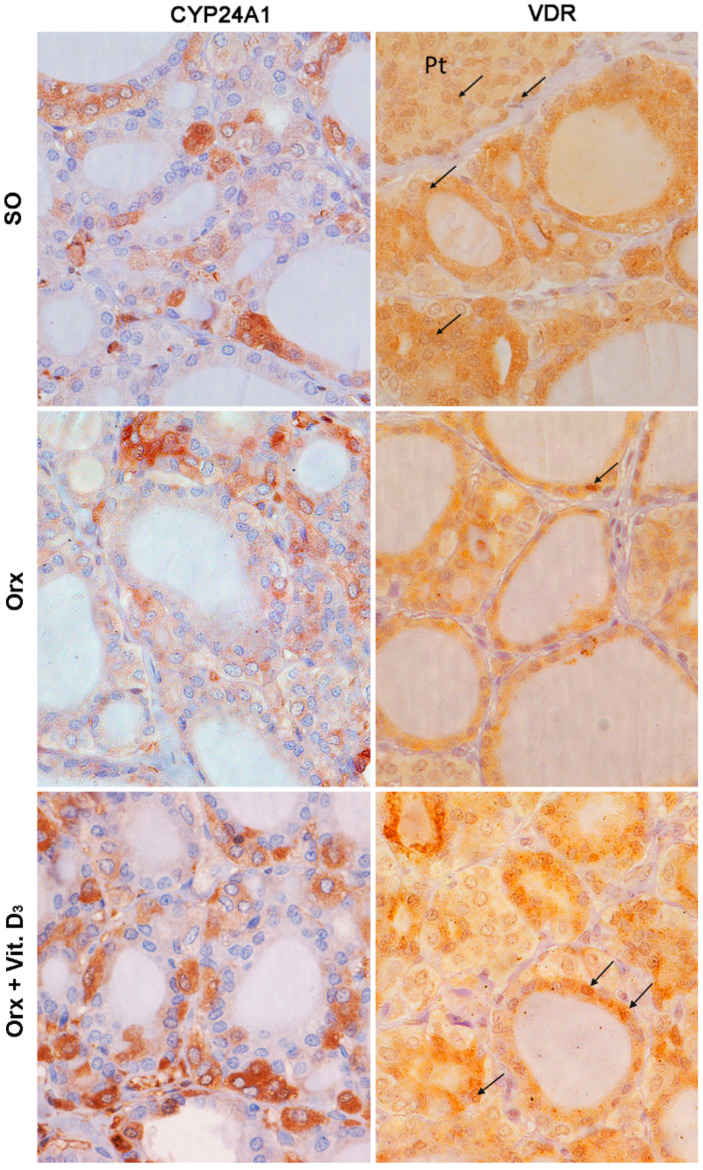
Representative micrographs of immunohistochemical staining showing localization of 24-hydroxylase (CYP24A1) and vitamin D receptor (VDR; arrows point to VDR-immunopositive nuclei) in the thyroid of sham-operated (SO), orchidectomized (Orx), and orchidectomized cholecalciferol-treated (Orx + Vit. D_3_) rats. Pt, parathyroid gland.

**Figure 6:**
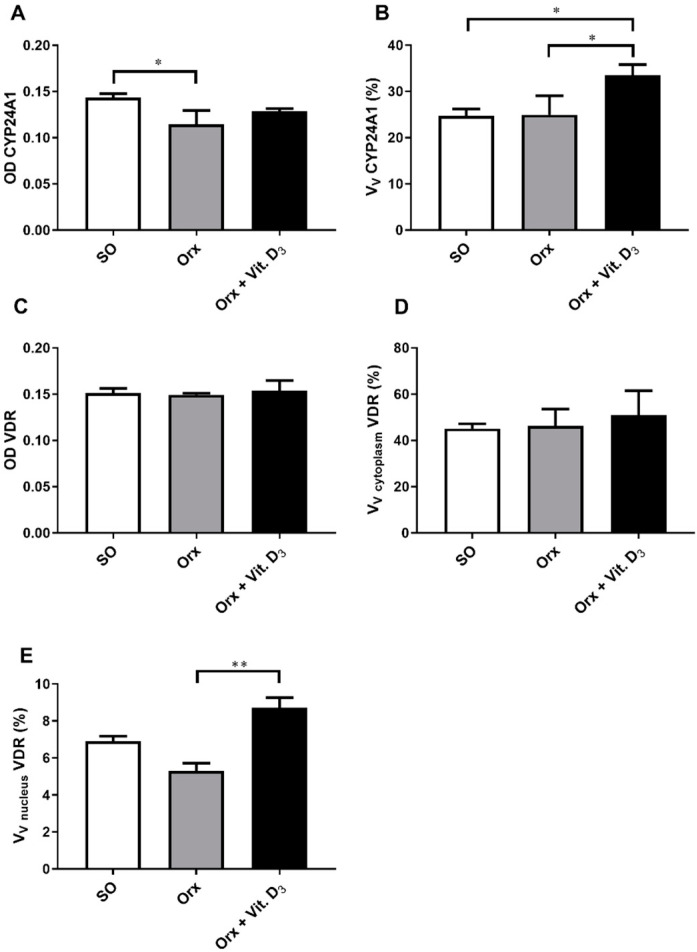
Optical density (OD) of CYP24A1 immunopositivity (**A**), the relative volume density of CYP2R1-immunopositive cells (Vv CYP2R1; %; (**B**)), the optical density (OD) of VDR immunopositivity (**C**), the relative volume density of vitamin D receptor in the cell cytoplasm (Vv cytoplasm VDR; %; (**D**)) and nucleus (V_V_ nucleus VDR; %; (**E**)) in the thyroid of sham-operated (SO), orchidectomized (Orx) and orchidectomized cholecalciferol-treated (Orx + Vit. D_3_) rats. All values are means ± SD (*n* = 6); *, *p* < 0.05; **, *p* < 0.01.

Immunohistochemical evaluation of expression of CYP24A1 in the thyroid gland confirmed its presence in the cytoplasm of thyroid cells (Figure 5). Aside from pale immunostaining present in thyrocytes, a subpopulation of intensely stained CYP24A1-immunopositive cells, located between or adjutant to follicles, with large light blue nuclei, was clearly distinguishable in the thyroid tissue (Figure 5). OD for CYP24A1 in the thyroid was 20% lower (*p* < 0.05) in the thyroid of the Orx group compared with the SO group (Figure 6A). Morphometric analysis revealed that the relative volume density of CYP24A1-immunopositive cells in the thyroid gland was significantly increased in the Orx + Vit. D_3_ group by 34% (*p* < 0.05) and 36% (*p* < 0.05) compared to Orx and SO groups, respectively (Figure 6B). 

High intensity of VDR immunostaining was mainly present in the cytoplasm of rat follicular thyroid cells, while it was pale in parafollicular C cells, more similar to the cytoplasmic immunostaining of positive control parafollicular cells (Figure 5). On the other hand, the presence of VDR-immunopositive nuclei was rare regardless of cell type, in contrast to parathyroid cells, the positive control of IHC staining (at the same histological section) in which IHC expression of VDR in nuclei was more intense compared with the cytoplasm (Figure 5). No difference in VDR IHC signal intensity was detected between the experimental groups (Figure 6C). The relative volume density of VDR-immunopositive cells with cytoplasmic IHC expression was not significantly changed upon Vit. D_3_ treatment (Figure 6D). However, V_V_ of VDR-immunopositive nuclei increased in the Orx + Vit. D_3_ group by 60%, *p* < 0.01, compared with the V_V_ indicated intensified utilization of stored colloid and release of thyroid hormone in the blood Orx group (Figure 6E).

**Figure 7:**
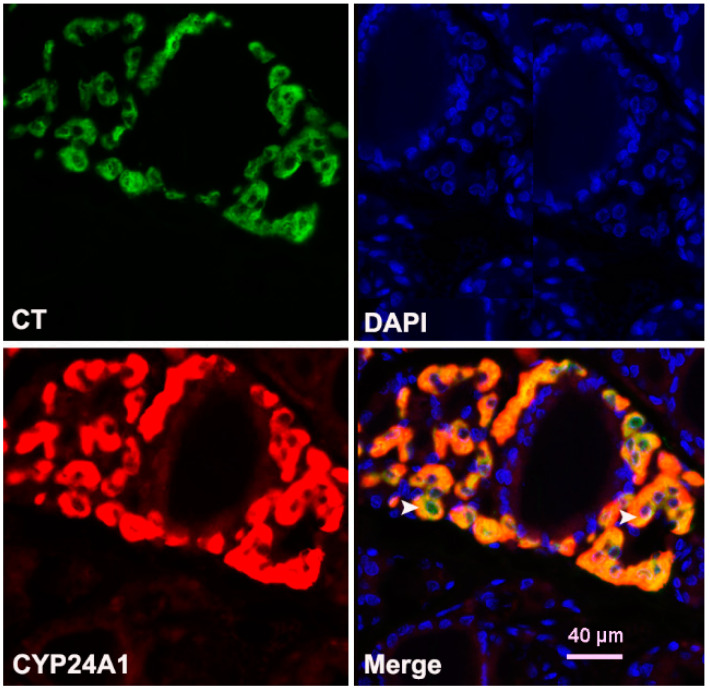
Double-immunofluorescence demonstrating colocalization of CYP24A1 and calcitonin (CT) in the thyroid of Orx + Vit. D_3_ rats. CYP24A1 (red fluorescence) was highly expressed by CT-containing cells (green fluorescence). Colocalization of CYP24A1 to CT-producing cells is depicted by yellow/orange color and accentuated by white arrowheads.

Colocalization analysis confirmed that strong CYP24A1 immunoreactivity colocalized with calcitonin (Figure 7).

## 4. Discussion

Vitamin D is an important and essential nutrient for bone health. Maintaining higher bone mineral density in the elderly can reduce the fracture risk, which may be achieved by pharmacological and dietary intervention [32]. Moreover, a normal euthyroid state is essential for maintenance of adult bone structure and strength [21]. There is no available information about how vitamin D treatment affects thyroid morphology and function in the context of osteoporosis. 

In this study, we measured the concentration of total 25(OH)D as the best biochemical marker for nutritional vitamin D status in humans [33,34] and the most abundant metabolite of this hormone in the blood of rats as well [35]. A dose of 5 µg or 200 IU/kg of rat b.m. was calculated using allometric scaling and corresponds to a therapeutic cholecalciferol dose of 6500 IU/day in humans. The Endocrinology Society guidelines recommend 6000 IU of vitamin D daily for 8 weeks for people who, in addition to vitamin D deficiency, suffer from osteoporosis and other metabolic disorders [33]. Vit. D_3_ treatment tripled the 25(OH)D level in serum in comparison with Orx rats, while this level was quadrupled compared with the SO group. Bearing in mind that CYP2R1 (the enzyme that catalyze first step in activation of Vit. D_3_) is expressed to the highest extent in the liver and testicles [36], this difference seems to be a logical consequence of testicular removal. 

Histological examinations of the thyroid gland revealed a microfollicular structure in Orx + Vit. D_3_ group that was more pronounced in comparison with the Orx group. Our previous research on the effect of orchidectomy in middle-aged rats revealed a decreased volume density of the colloid in the thyroids, decreased activities of deiodinase type 1 in the liver, and deiodinase type 2 in the pituitary. The changes obtained indicated compensation/adaptation of the examined tissues to hypothyroidism, despite serum T_4_ and TSH remaining unchanged [27]. Vit. D_3_ treatment of Orx rats further decreased relative volume density of colloid, which indicates lower synthesis of thyroglobulin in comparison with its utilization, resorption, and probable thyroid hormone release. In line with this was the ultrastructural finding of an increased presence of large electron dense lysosomes in the thyrocytes [37] in Orx + the Vit.D_3_ group. The elevated number of lysosomes may be due to increased efflux of iodide and iodothyronines, which therefore could lead to decreased levels of colloid in the follicular lumen [38]. A slightly increased efflux despite lower iodide uptake was demonstrated in FTRL-5 cells exposed to 1,25(OH)_2_D by Berg et al. [18].

Moreover, Vit. D_3_ treatment lowered immunostaining intensity of TPO and the expression of NIS at the basolateral membrane, which was more prominent in the cytosol. This results indicate altered iodide trapping and thyroid hormone synthesis [39]. In line with this assumption, calcitriol treatment attenuated iodide porter number and iodide uptake in the rat thyroid FRTL-5 cell line [40,41]. In this context, the somewhat enlarged relative thyroid weight of the Orx + Vit. D_3_ group compared with weights obtained for the SO group, although non-significant, may be worth further investigation in subsequent studies of higher power. Increase of thyroid weight, most often associated with iodine deficiency, is a compensatory mechanism to overcome the decreased hormone synthesis [42].

The results of this study confirmed immunohistochemical staining of CYP24A1 enzyme and VDR in the rat thyroid gland. In this respect, the thyroid responded to vitamin D_3_ treatment in a fashion similar to the classical vitamin D target tissues. The original finding of this study is finding that nuclear VDR were more pronounced in the thyrocytes, while the subpopulation of parafollicular C cells exerted stronger CYP24A1 immunostaining. The Western blot method is considered more reliable technique for gathering quantitative data in comparison with IHC, which is a limitation of this study. However, keeping in mind the difference in expression of examined proteins between follicular and parafollicular cells, as well as the close proximity of parathyroid glands (difficult to remove during tissue homogenization for Western blot analysis, but excellent to serve as the positive control tissue on the same slide for IHC), we decided to use this method. 

Examinations of IHC expression of CYP24A1 in our study was inspired by the previous report of Bennett et al. [16], who confirmed that both normal and various types of thyroid cancer cells expressed key proteins involved in Vit. D metabolism and signaling, but that expression of CYP 24A1 was lower in cancer cell lines. Orchidectomy reduced the intensity of the Cyp24A1 IHC signal, while subsequent treatment of Orx rats with Vit D increased the volume density of CYP24A1-immunopositive cells. The results obtained are in line with changes in serum 25(OH)D in Orx and Orx + Vit.D_3_ rats. Previous reports on the expression of catabolic enzyme CYP24A1 confirmed that its expression is highly induced by 1,25(OH)_2_D [34,43]. 

The original finding of this research is also identification of a subpopulation of intensively CYP24A1-immunostained cells in rat thyroids, with increased volume percentage in Orx + Vit D_3_ animals in comparison with corresponding controls. Double IFC staining results confirmed the presence of parafollicular CT-immunopositive cells co-expressing strong CYP24A1 signal in thyroid tissue. Calcitonin concentration in serum was not significantly altered in Orx + Vit.D_3_ compared to the Orx group, being similarly lower compared with SO controls. Other researchers have shown that Vit D treatment inhibits CT secretion in rodents, although the effect on cell proliferation was contradictory [12,13,44]. Calcitonin secretion is stimulated in conditions of elevated serum calcium concentration and this hormone protects against the development of hypercalcemia [45]. Further study more focused on morphofunctional features of C cells, calcium/phosphorus and bone homeostasis in our model will allow more detailed understanding of the effects of Vit. D_3_ on this thyroid endocrine cell subpopulation. 

Nuclear VDR immunostaining has not been significantly decreased in the thyroid of Orx group in comparison with SO animals. It is tempting to speculate that subtle, non-significant decrease in nuclear VDR expression in the thyroid of Orx middle-aged group may be of a higher magnitude upon orchidectomy of young adult animals, keeping in mind higher testicular production of 25-(OH)D in young adults [36]. In contrast to middle-aged animals, orchidectomy of young adults induced more prominent changes in thyroid economy and reduced TSH level significantly [30]. Further study of VDR-mediated signaling in orchidectomized young adults is needed to examine its possible role in disturbance of thyroid homeostasis at both the level of thyroid and the pituitary.

Immunohistochemical evaluation of VDR expression revealed more prominent nuclear immunostaining in thyrocytes of Orx + Vit. D_3_ group in comparison with corresponding controls. According to Clinckspoor et al. [12], altered 1,25(OH)_2_D-VDR signaling does not influence normal thyroid development nor the function of thyrocytes in rodents. However, our results indicate that the thyroid responded to Vit. D treatment as a classical target organ, with great ability to compensate these changes and maintain thyroid hormone balance in serum. In the rat thyroid FRTL-5 cell line, calcitriol attenuated both TSH-stimulated cAMP production and the effects of cAMP [46,47], while those effects were mainly mediated by genomic VDR-signaling [18]. 

## 5. Conclusions

In this study, we showed—for the first time—that vitamin D_3_ treatment of Orx middle-aged rats, our model of osteoporosis, changed thyroid morphology in a way that indicates an intensified colloid resorption and hormone release, which was probably compensated by lower hormone synthesis, as circulatory levels of T_4_ and TSH remained unchanged. The thyroid responded to vitamin D_3_ treatment in a fashion similar to classical vitamin D target tissues, and increased nuclear VDR in follicular cells indicates direct, TSH-independent, action of vitamin D. On the other hand, immunohistochemical staining of vitamin D catabolic enzyme CYP24A1 was more intense in parafollicular C cells, indicating its prominent expression in response to Vit. D in this thyroid endocrine cell population. The obtained results suggest that indirect effect of vitamin D on bone, through fine regulation of thyroid function, is small. 

## Figures and Tables

**Table 1:** List of primary and secondary antibodies used in IHC/IFC staining.

Name	Manufacturer	Cat. Number	Origin	Dilution
TPO	Santa Cruz, Italy	sc-376876	Mouse	1:400
NIS	Acris, Germany	EUD4101	Rabbit	1:600
Tg	Dako, Denmark	A0251	Rabbit	1:500
CYP24A1	Santa Cruz Biotech Inc., Italy	sc-66851	Rabbit	1:100
VDR	Abcam, UK	Ab 3508	Rabbit	1:1000
CT	Dako, Denmark	A576	Rabbit	1:300
Anti-mouse, HRP labeled	Abcam, UK	Ab6820	Donkey	1:200
Anti-rabbit, HRP labeled	Dako, Denmark	P0399	Swine	1:200

**Table 2:** Absolute and relative thyroid weight, body mass.

Group	SO	Orx	Orx + Vit. D_3_	Statistical Significance
Absolute thyroid weight (mg)	40.00 ± 7.69	44.33 ± 5.43	41.00 ± 5.90	n.s.
Relative thyroid weight	0.049 ± 0.007	0.059 ± 0.009	0.063 ± 0.01	n.s. vs. Orx *p* = 0.08 vs. SO
Body mass (g)	683.3 ± 52.8	675.0 ± 63.5	665.0 ± 68.9	n.s.

Results are mean ± SD, *n* = 6.
